# Impact of Oral Health Training on Primary Health Care Workers in Gandaki Province of Nepal: A Pilot Study

**DOI:** 10.1155/ijod/5499705

**Published:** 2025-10-07

**Authors:** Krishna Subedi, Umesh Parajuli, Prakash Raj Sharma, Ramesh Prasad Adhikari

**Affiliations:** ^1^Department of Community Dentistry, Gandaki Medical College Teaching Hospital and Research Center, Pokhara, Nepal; ^2^Department of Orthodontics, Gandaki Medical College Teaching Hospital and Research Center, Pokhara, Nepal; ^3^Gandaki Province Health Training Center, Pokhara, Nepal

**Keywords:** health care providers, impact, oral health, primary, training

## Abstract

**Introduction:**

This study was done to assess the impact of oral health training on primary health care (PHC) workers in Gandaki Province, Nepal.

**Methods:**

This quasi-experimental study was conducted among 24 PHC providers (auxiliary health workers [AHWs], and public health inspectors [PHIs]) attending the oral health training from 11 districts of Gandaki province. A non-randomized sampling technique was used. All participants present during the training session who provided written informed consent were included in the study. Dental experts provided training to the participants for 6 days, the last week of April 2025. The method of training uses a PowerPoint presentation, different tooth models and brushing models, diagnostic instruments, fluoride varnish, and videos. Oral health training was evaluated by assessing the improvements in oral health knowledge (OHK) and practice. There were 30 questions for assessing OHK and 8 questions for oral health practices. After completion of the study, data were entered in a Microsoft Excel Sheet version 2013 and analyzed using R 4.3.2 software. The level of significance was set at *p*  < 0.05. Paired *t*-test was used to compare the baseline and the end of training for knowledge scores.

**Results:**

The participants' mean age was 32.04 ± 7.54 years, ranging from 21 to 44 years. The overall knowledge was improved by 55.25% from 16.83 ± 2.82 to 26.13 ± 2.38 (*p*  < 0.001, Cohen's *d* = 3.54) after training, a statistically significant difference. Before the training, most participants had good oral hygiene practices. There was a slight improvement in these practices after the training. At baseline, 35.29% (6 participants) were using dental floss among those using interdental aids, while after training, this percentage increased to 86.36% (19 participants).

**Conclusions:**

The study concluded that oral health training effectively improved knowledge and practices related to oral health.

**Trial Registration:**

Clinical Trial Registry India (CTRI) identifier: CTRI/2024/04/065782 (REF/2024/04/082334)

## 1. Introduction

Oral diseases cover a spectrum of conditions, including dental caries, periodontal disease, tooth loss, oral cancer, oro–dental trauma, noma, and developmental disorders like cleft lip and palate. Oral diseases are among the most common noncommunicable diseases worldwide, with a disproportionate impact on vulnerable and disadvantaged populations [1]. Oral diseases are highly preventable [2], and oral health care should be integrated into primary healthcare services [3].

“Primary health care (PHC)” is the first level of contact where essential health care services are provided to an individual [4]. The World Health Organization (WHO) recommends achieving universal health coverage (UHC), and oral health services should be accessible and easily used by all people [5]. Oral health care should be integrated into PHC and ensure related financial protection and essential supplies in PHC [5]. Integration of essential oral health care into PHC and UHC is one of the six strategic objectives of the Global strategy on oral health [6].

Integrating oral health care into primary care is grounded in evidence showing the close connection between oral health and overall health. Coordinating care between these areas is vital for maintaining overall health. Primary care settings offer a valuable opportunity to address oral health by conducting risk assessments, screenings, and preventive measures, and providing guidance, counseling, and referrals to dental professionals [7]. Integrating oral health care into primary care can be achieved through interprofessional education, collaborative practice, closed-loop referral systems, partnerships between public and private sectors, and the inclusion of dental professionals within primary care teams [3].

The Nepalese Constitution recognizes health as a basic human right. Health posts are Nepal's first institutional contact point for basic health services [8]. They are particularly run by auxiliary health workers (AHWs; health assistants [HAs], community medical assistants, auxiliary nurse midwives) with other helping staff. There are no dentists in health posts. Therefore, it is necessary to provide oral health-related training to these staff unless dentists are recruited into the health posts. The success of the training should be evaluated before implementing it. To the best of the author's knowledge, no studies of this nature in Nepal have been published to date. Therefore, this study was done to assess the impact of oral health training on PHC workers in Gandaki Province, Nepal.

## 2. Methods

### 2.1. Study Design, Setting, and Eligibility Criteria for Participants

This was a quasi-experimental study conducted among PHC providers (AHWs and public health inspectors [PHIs]) attending the oral health training. A total of 24 healthcare workers from all districts of Gandaki Province participated in the study. In Gandaki Province, there were a total of 18 public health officers/health education administrative officers and 67 paramedics (also known as AHWs, which include nursing staff, lab staff, and pharmacy) and 81 nursing staff [9]. There are a total of 11 districts in the province. Three each from Baglung and Myagdi, and two each from the remaining districts of Gandaki province were selected. All the participants who were present during the training session and who had provided written informed consent were included. The training was provided by the Health Training Center, Pokhara, Nepal. All the expenses of the training were covered by the training center.

The Health Training Center of Gandaki Province sent invitation letters to all health offices across the province's districts, inviting health workers who were working at the local level to participate. Selection criteria included individuals working as permanent primary health workers (PHIs, HAs, senior community medical assistants, community medical assistants, or AHWs). They were instructed to choose three participants from both Baglung and Myagdi and two participants from each of the remaining districts in the Gandaki province. Participants were selected by their respective district health offices.

### 2.2. Ethical Approval and Consent

The study was conducted following the principles of the Declaration of Helsinki. Ethical approval for this study was obtained from the Institutional Review Committee of Gandaki Medical College (Reference Number: 37/080/081).

### 2.3. Interventions

Oral health training included topics like oral anatomy, specialization in dentistry, dental caries and its sequelae, teeth hypersensitivity, dentoalveolar abscess, space infection, fluoride, fluoride varnish and its application, gingivitis and periodontitis, brushing and flossing methods, common oral lesion, tobacco and oral cancer, systemic disease and trigeminal neuralgia, common pediatric problems, eruption disorder, traumatic problems, malocclusion, TMJ dislocation, role of diet on oral health. Various dental experts (recommended by the training center) provided training to the participants in the above-mentioned topics for 6 days, in the last week of April 2025. The method of training used a PowerPoint presentation, different tooth models and brushing models, diagnostic instruments (mouth mirror, explorer, a pair of tweezers), fluoride varnish, and videos.

### 2.4. Evaluation of Intervention

Oral health training was evaluated by assessing the improvements in oral health knowledge (OHK) and practice.

### 2.5. Overall OHK and Practice Assessment

There were a total of 30 questions for assessing OHK. There were eight questions for oral health practices. The questions were developed by the authors based on the topics to be covered during the training process, which are provided in Appendix A. Each of the 30 multiple-choice questions had a single correct answer. All questions had a binary outcome coded as one for correct and zero for incorrect. Every correct answer in baseline and after 6 days of training was scored as 1, and wrong answers were scored as zero. An overall composite score was then created by adding the individual scores on each question. The highest possible score for OHK was 30 for each individual.

The highest possible score for oral health practices was 7 (question for oral health practices number 5. How often do you change your toothbrush? was not provided a score, as it was only taken at the baseline and not considered in the post-training questionnaire. As the study evaluated the change in practice within 6 days, changing the toothbrush could not be done within this period). The mean score of knowledge was then calculated and then compared between baseline and after training. The percentage change was calculated by subtracting the pretest percentage from the posttest percentage (100 × [baseline mean score-after training score]/baseline score).

There were three questions regarding their previous training related to oral health, any oral health services provided in their workplace, and what services they were providing in their workplace. This questionnaire was filled out at the baseline only.

The questionnaire was filled out by the participants. The researcher checked for the completeness of the questionnaire before collecting the filled questionnaires.

### 2.6. Validation of the Study Tool

The content validity of the questionnaire was done with five experts, and the content validity index was found to be more than 0.90.

### 2.7. Masking

No masking was performed as health education was provided to all participants, and there was no control group.

### 2.8. Statistical Analysis

After completion of the study, data were entered in a Microsoft Excel Sheet version 2013 and analyzed using R 4.3.2 software. The level of significance was set at *p* < 0.05. The Shapiro–Wilk test was used to check the normality of the data, as the sample is less than 50. A descriptive analysis was performed to summarize the socio–demographic characteristics. Descriptive statistics, including the mean and standard deviations, were computed for OHK. Paired *t*-test was used to compare the baseline and at the end of training for knowledge and practice scores. The McNemar test was used to compare the percentage of correct responses of OHK before training and after training.

## 3. Results

All participants completed the study with a 100% response rate and no loss to follow-up. The data distribution was assessed for normality using the Shapiro–Wilk test. Before training, the OHK scores exhibited normal distribution (Shapiro–Wilk test, *W* = 0.942, *p*-value = 0.176, as did the OHK scores after training (Shapiro–Wilk test, *W* = 0.958, *p*-value = 0.402).

The participants' mean age was 32.04 ± 7.54 years, ranging from 21 to 44 years. Half of the participants were under 30 years old. Two-thirds of the participants were male. Approximately half of the participants had completed HA education. Half of the participants worked as AHWs. Slightly over half of the participants had more than 5 years of work experience (Table 1).

None of the participants had received any prior training related to dental and oral health. More than three-fifths of the participants were involved in providing dental and oral health-related services in their respective areas. Slightly less than half of the participants offered dental counseling and referrals for treatment (Table 2).

**Figure 1:**
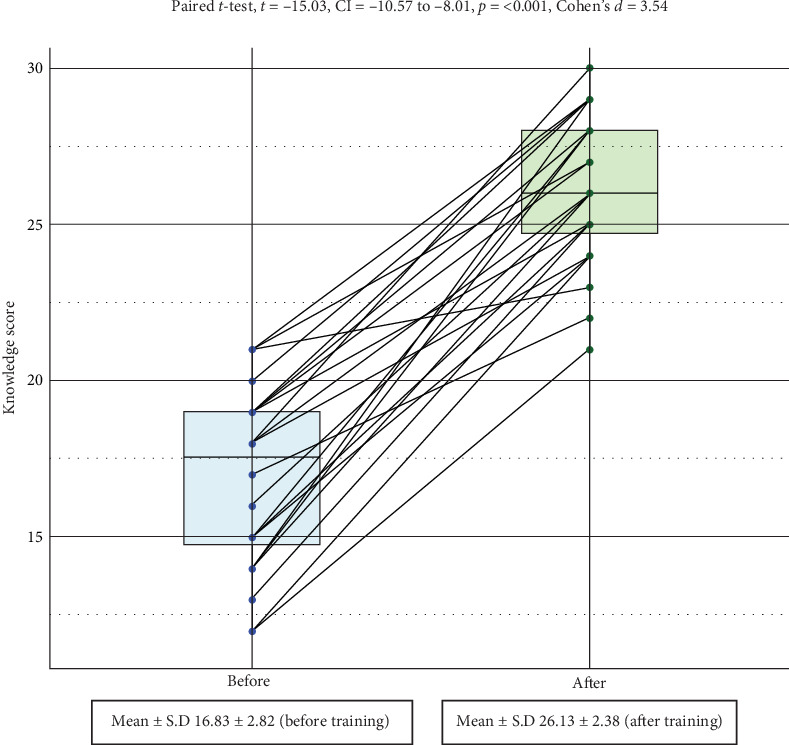
Paired line plot of mean knowledge of health workers before and after training (*N* = 24).

The mean OHK at the baseline was 16.83 ± 2.82. This increased by 55.25% to 26.13 ± 2.38 after training, a statistically significant difference (Figure 1).

Out of 30 questions, all participants provided the correct response to only one question, “What are the essential elements for tooth decay?” at baseline. The total responses to 14 questions (QN: 3, 5, 6, 7, 9, 11, 13, 19, 20, 21, 24, 28, 29, 30) showed statistically significant improvement after 6 days of training (Table 3).

Before the training, most participants had good oral hygiene practices. There was a slight improvement in these practices after the training. At baseline, 35.29% were using dental floss among those using interdental aids, while after training, this percentage increased to 86.36% (Table 4).

## 4. Discussion

### 4.1. OHK

This study demonstrated that oral health education effectively enhanced OHK among healthcare workers in Gandaki Province. The overall knowledge increased by 55.27%, from 16.88 ± 2.86 to 26.21 ± 2.44. No similar studies have been conducted. However, numerous studies have explored the impact of oral health training on nurses and other healthcare workers. A study conducted in India by Sandhya et al. [10] among primary healthcare workers at primary healthcare centers showed an overall increase in knowledge levels, with a mean difference of 12.56 ± 3.23, which aligns closely with our study findings. Similar results were observed in a study conducted by Bhagia et al. [11] in India.

A study done by Wu et al. [12] in China in 2020 showed that the mean knowledge was significantly improved from 6.58 ± 1.23 to 7.32 ± 0.70 among staff providing long-term care services. A study by Mohebbi et al. [13] in Iran on primary care physicians showed that oral health care knowledge was improved after intervention. Another study conducted in Nigeria by Oladayo et al. [14] among nurses and community health workers also demonstrated an increase in OHK after the training.

The findings from this study suggest that knowledge of oral health among primary healthcare workers can be improved through oral health education.

In rural areas where dental manpower is insufficient, preventive oral health education can be provided to healthcare workers. This enables them to share knowledge with those in need within their communities, aiding in the prevention of dental diseases to some extent.

### 4.2. Oral Health Practices

The majority of participants already had good oral hygiene practices before the training. The use of interdental aids increased from 70.83% to 91.67%. Before training, 35.29% of participants using interdental aids were using dental floss, which increased to 86.36% after training. Despite already having good oral hygiene practices before training, there was an improvement after training within a very short period of time. This improvement may be attributed to increased OHK. However, the long-term impact on oral hygiene practices should be evaluated.

## 5. Strengths and Limitations

Health workers from all districts of Gandaki province were included in the study, which enhances its representativeness. However, the use of convenience sampling limits the generalizability of the findings. Additionally, the study did not utilize a separate control group. Social desirability bias is also a potential concern. Furthermore, the study was also of shorter duration, and long-term knowledge retention and practice were not evaluated. Therefore, the results should be interpreted as an assessment of immediate recall rather than long-term retention.

## 6. Conclusions

The study concluded that oral health education effectively improves OHK and oral hygiene practices in the short term. However, a long-term evaluation of its impact is necessary. If proven effective in the long term, it should be fully implemented, providing oral health education to all healthcare providers in Gandaki Province.

## Figures and Tables

**Table 1:** Socio–demographic details of the participants (*N* = 24).

Variables	Frequency (*N*)	Percentage (%)
Age (in years)		
21–30	12	50.0
31–40	8	33.33
>40	4	16.67
Sex		
Male	16	66.67
Female	8	33.33
Education		
CMA	9	37.50
HA	11	45.83
BPH	4	16.67
Post		
Auxiliary health workers (AHWs) (community medical assistants/health assistants)	12	50.00
Senior auxiliary health workers (AHWs)/senior community medical assistants	6	25.00
Public health inspector	6	25.00
Work experience (in years)		
<2 years	6	25.0
2–5 years	5	20.83
>5 years	13	54.17

**Table 2:** Responses to previous training related to oral health, any oral health services provided in their workplace (*N* = 24).

Question	Responses	
	Yes *N* (%)	No *N* (%)
1 Have you taken any dental and oral health-related training?	0	24 (100.0%)
2. Do you provide dental and oral health-related services at your health post?	20 (83.33%)	4 (16.67%)
3. If yes, please specify		

	**Responses**	***N* (%)**

	Counseling and referral	11 (45.83%)
	Counseling and prescribing medicine	7 (29.17%)
	Counseling, prescribing medicine, and referral	2 (8.33%)

**Table 3:** Comparison of knowledge of health workers before and after training using the McNemar test (*N* = 24).

Questions	Correct responses	At baseline *N* (%)	After training *N* (%)	*p*-Value
1. What is the outer part of the crown made of?	Enamel	20 (83.33%)	24 (100.0%)	0.125
2. According to the FDI system, 21 refers to which tooth?	Permanent left central incisor	8 (33.33%)	16 (66.67%)	0.057
3. What is the name of the dental expert manpower who corrects the malaligned teeth using braces?	Orthodontist	14 (58.33%)	22 (91.67%)	**0.021**
4. How many milk teeth are present in a child?	20	23 (95.83%)	24 (100.0%)	1.00
5. What is the name of the teeth that appear in the mouth at birth?	Natal teeth	2 (8.33%)	22 (91.67%)	**<0.001**
6. Do you think it is necessary to clean the gum pads before the eruption of teeth?	Yes	16 (66.67%)	23 (95.83%)	**0.016**
7. Do you think that prolonged breastfeeding and bottle feeding during the night will cause tooth decay?	Yes	11 (45.83%)	24 (100.0%)	**<0.001**
8. Should the decay in the milk tooth be treated?	Yes	17 (70.83%)	23 (95.83%)	0.070
9. Early childhood caries is caused by which microorganism?	*S. mutans*	4 (16.67%)	18 (75.0%)	**<0.001**
10. What are the essential elements for tooth decay?	Both A and B	24 (100.0%)	24 (100.0%)	NA
11. Pain in dental caries occurs after the involvement of?	Pulp	15 (62.50)	22 (91.67%)	**0.039**
12. What are the measures of prevention in dental caries?	All of the above	23 (95.83%)	24 (100.0%)	1.00
13. Which of the following teeth is most likely to be infected with caries?	First molar	8 (33.33%)	15 (62.50%)	**0.039**
14. What is fluoride?	A chemical substance in water that prevents dental caries	18 (75.00%)	22 (91.67%)	0.219
15. Which of the following habits is not related to malocclusion?	Crawling	16 (66.67%)	18 (75.0%)	0.754
16. Up to what age can the teeth be corrected using braces?	Up to any age if supporting structures of teeth like gums and alveolar bone are healthy	20 (83.33%)	20 (83.33%)	1.00
17. Are people with diabetes at a higher risk of having gum disease?	Yes	20 (83.33%)	24 (100.0%)	0.125
18. What can be done if the tooth is mobile due to trauma/injury?	Refer to dentists for splinting	20 (83.33%)	22 (91.67%)	0.688
19. Storage media for avulsed tooth	All of the above	1 (4.17%)	23 (95.83%)	**<0.001**
20. What is the problem of teeth grinding due to using abrasive toothpaste or a hard brush?	Abrasion	5 (20.83%)	19 (79.17%)	**<0.001**
21. How long should you wait before brushing after consuming acidic foods or drinks?	After 30 min–1 h	11 (45.83%)	20 (83.33%)	**0.022**
22. What is/are the methods to treat hypersensitive teeth?	All of the above	21 (87.50%)	23 (95.83%)	0.625
23. What is the first sign of gingivitis?	Swollen gums that bleed	17 (70.83%)	21 (87.50%)	0.125
24. Trigeminal neuralgia is characterized by	Sharp, excruciating pain of short duration	4 (16.67%)	21 (87.50%)	**<0.001**
25. What is the problem with temporomandibular Joint dislocation?	Unable to close mouth	13 (54.17%)	22 (91.67%)	0.004
26. Pericoronitis is usually seen in which teeth	Third molar	14 (58.33%)	17 (70.83%)	0.453
27. Which of the following is not a symptom seen in the mouth of a diabetic patient?	Whitening of teeth	13 (54.17%)	16 (66.67%)	0.549
28. Which trimester of pregnancy is safer to undergo dental treatment?	Second trimester	10 (41.67%)	19 (79.17%)	**0.004**
29. Which vitamin deficiency leads to beefy red tongue?	Vitamin B	9 (37.50%)	18 (75.0%)	**0.004**
30. Which of the following diseases is caused by the use of betel nut/areca nut and betel nut products?	Oral submucous fibrosis	8 (33.33%)	21 (87.50%)	**0.001**

*Note:* The bold indicates statistically significant *p*-values.

**Table 4:** Oral hygiene practices before and after training (*N* = 24).

Questions	Responses	Responses at baseline	Responses after training
1. How do you clean your teeth?	Toothbrush and toothpaste	24 (100.0%)	24 (100.0%)

2. How often do you brush your teeth daily?	Twice daily after each main meal	21 (87.50%)	23 (95.83%)
	Once a day in the morning	3 (12.50%)	0
	Once a day in the evening	0	1 (4.17%)

3. Do you brush before meals or after meals?	After meal	22 (91.67%)	24 (100.0%)
	Before meal	2 (8.33)	—

4. What type of toothbrush bristle do you use?	Soft	17 (70.83%)	20 (83.33%)
	Medium	5 (20.83%)	1 (4.17%)
	Ultra-soft	1 (4.17%)	3 (12.50%)
	Hard	1 (4.17%)	0

5. How often do you change your toothbrush?	Every 3–4 months	10 (41.67%)	NA
	Every 2 months	9 (37.50%)	—
	When bristles flare out	3 (12.50%)	—
	Every month	1 (4.17%)	—
	Every 6 months	1 (4.17%)	—

6. Did you clean/rinse your teeth last time after eating sweets or chocolates?	Yes	22 (91.67%)	24 (100.0%)
	No	2 (8.33%)	0

7. Do you clean your tongue?	Yes	21 (87.50%)	24 (100.0%)
	No	3 (12.50%)	0

8. Do you also clean between the teeth?	Yes	17 (70.83%)	22 (91.67%)
	No	7 (29.17%)	2 (8.33%)

8.1 If yes, what do you use to clean?	Dental floss	6 (35.29%)	19 (86.36%)
	Interdental brush	3 (17.65%)	3 (13.64%)
	Toothpick	8 (47.06%)	0

## Data Availability

The data will be made available upon reasonable request to the corresponding author (Krishna Subedi).

## Nomenclature

AHW: Auxiliary health worker
CMA: Community medicine assistant
HA: Health assistant
OHK: Oral health knowledge
WHO: World Health Organization
UHC: Universal health coverage.

## Appendix A

**Questionnaire in English**

Age (in years):  Sex: male/female   Education:

Post:       Working experience as a health care worker (in years):

**Questionnaire related to previous training related to oral health, and any oral health services provided in their workplace at the professional level (this information should be filled only at the baseline).**

**1. Have you taken any dental and oral health-related training?**

A. Yes B. No

**2. Do you provide dental and oral health-related services at your health post?**

A. Yes B. No

**3. If yes, please specify:**

A. Counseling and referral B. Counseling and prescribing medicine C. Others (specify):

**Oral health knowledge**

**Please circle the correct answer.**

**1. What is the outer part of the crown made of?**

A. Dentin B. Cementum C. Enamel D. Pulp

**2. According to the FDI system, 21 refers to which tooth?**

A. Permanent right central incisor B. Permanent left central incisor

C. Permanent left lateral incisor D. Permanent left first premolar

**3. What is the name of the dental expert manpower who corrects the malaligned teeth using braces?**

A. Orthodontist B. Prosthodontist C. Endodontist D. Maxillofacial surgeon

**4. How many milk teeth are present in a child?**

A. 18 B. 20 C. 28 D. 32

**5. What is the name of the teeth that appear in the mouth at birth?**

A. Neonatal teeth B. Natal teeth C. Molar D. Mesiodens

**6. Do you think it is necessary to clean the gum pads before the eruption of teeth?**

A. Yes B. No

**7. Do you think that prolonged breastfeeding and bottle feeding during the night will cause tooth decay?**

A. Yes B. No

**8. Should the decay in the milk tooth be treated?**

A. Yes B. No

**9. Early childhood caries is caused by which microorganism?**

A. *S. mutans *B. *Lactobacillus *C. *P*. *gingivalis *D. *Candida albicans*

**10. What are the essential elements for tooth decay?**

A. Fermentable bacterial substrate, sugar B. Bacterial Plaque

C. Both A and B D. Dirty hands

**11. Pain in dental caries occurs after the involvement of?**

A. Enamel B. Dentin C. Pulp D. All of the above

**12. What are the measures of prevention in dental caries?**

A. Brushing teeth using fluoridated toothpaste once in the morning after food and before bed at night

B. Mouthrinse after eating sweets, sticky, and any foods

C. Using dental floss to clean debris in between teeth

D. All of the above

**13. Which of the following teeth is most likely to be infected with caries?**

A. First molar B. Second molar C. Canine D. Incisors

**14. What is fluoride?**

A. A substance that purifies water

B. A substance that improves the taste of food

C. A chemical substance in water that prevents dental caries

D. All

**15. Which of the following habits is not related to malocclusion?**

A. Thumb sucking B. Tongue thrusting C. Crawling D. Mouth breathing

**16. Up to what age can the teeth be corrected using braces?**

A. Up to 30 years

B. Up to 40 years

C. Up to any age if supporting structures of teeth, like gums and alveolar bone, are healthy

D. Possible only in children

**17. Are people with diabetes at a higher risk of having gum disease?**

A. Yes B. No C. I do not know

**18. What can be done if the tooth is mobile due to trauma/injury?**

A. Nothing, leave it as it is B. Refer to dentists for splinting

C. Extraction of a tooth D. Recommend brushing vigorously

**19. Storage media for an avulsed tooth is/are**

A. Put into own mouth/socket B. Water C. Cow's milk D. All of the above

**20. What is the problem of teeth grinding due to using abrasive toothpaste or a hard brush?**

A. Attrition B. Abrasion C. Erosion D. Abfraction

**21. How long should you wait before brushing after consuming acidic foods or drinks?**

A. No need to wait, you can brush immediately B. After 10 min

C. After 20 min D. After 30 min to an hour

**22. What is/are the methods to treat hypersensitive teeth?**

A. Removal of causes B. Using fluoridated toothpaste

C. Restore cavity in tooth D. All of the above

**23. What is the first sign of gingivitis?**

A. Oral ulcers B. White gums C. Swollen gums that bleed D. I do not know

**24. Trigeminal neuralgia is characterized by**

A. Paralysis of one side of the face

B. Uncontrollable twitching of muscles

C. Sharp, excruciating pain of short duration

D. Prolonged episodes of pain on one side of the face

**25. What is the problem with temporomandibular Joint dislocation?**

A. Unable to open mouth B. Unable to close mouth

C. Pain in chewing D. Difficulty in speaking

**26. Pericoronitis is usually seen in which teeth?**

A. Third molar B. Second molar C. Canine D. Premolar

**27. Which of the following is not a symptom seen in the mouth of a diabetic patient?**

A. Dry mouth B. Burning mouth

C. Delayed in wound healing D. Whitening of teeth

**28. Which trimester of pregnancy is safer to undergo dental treatment?**

A. First trimester B. Second trimester

C. Third trimester D. First and second trimester

**29. Which vitamin deficiency leads to beefy red tongue?**

A. Vit. A B. Vit. B C. Vit. C D. Vit. K

**30. Which of the following diseases is caused by the use of betel nut/areca nut and betel nut products?**

A. Oral leukoplakia B. Oral submucous fibrosis

C. Oral lichen planus D. Erythroplakia

**Oral hygiene practices (at personal level)**

**Please circle the appropriate response.**

**1. How do you clean your teeth?**

A. Toothbrush and toothpaste B. Toothbrush and toothpowder C. Others (specify):

**2. How often do you brush your teeth daily?**

A. Once a day in the morning B. Once a day in the evening

C. Twice daily after each main meal D. More than two times E. Not daily

**3. Do you brush before meals or after meals?**

A. Before meal B. After meal

**4. What type of toothbrush bristle do you use?**

A. Very soft B. Soft C. Medium D. Hard E. I do not know

**5. How often do you change your toothbrush?**

A. Every month B. Every 2 months C. Every 3–4 months

D. Every 6 months E. Every year F. When bristles flare out

**6. Did you clean/rinse your teeth last time after eating sweets or chocolates?**

A. Yes B. No

**7. Do you clean your tongue?**

A. Yes B. No

**8. Do you also clean between the teeth?**

A. Yes B. No

**8.1 If yes, what do you use to clean?**

A. Dental floss B. Toothpick C. Interdental brush D. Others (specify):
